# Piperaquine Pharmacokinetics during Intermittent Preventive Treatment for Malaria in Pregnancy

**DOI:** 10.1128/AAC.01150-20

**Published:** 2021-02-17

**Authors:** Palang Chotsiri, Julie R. Gutman, Rukhsana Ahmed, Jeanne Rini Poespoprodjo, Din Syafruddin, Carole Khairallah, Puji B. S. Asih, Anne L’lanziva, Kephas Otieno, Simon Kariuki, Peter Ouma, Vincent Were, Abraham Katana, Ric N. Price, Meghna Desai, Feiko O. ter Kuile, Joel Tarning

**Affiliations:** a Mahidol-Oxford Tropical Medicine Research Unit, Faculty of Tropical Medicine, Mahidol University, Bangkok, Thailand; b Malaria Branch, Division of Parasitic Diseases and Malaria, Center for Global Health, Centers for Disease Control and Prevention, Atlanta, Georgia, USA; c Department of Clinical Sciences, Liverpool School of Tropical Medicine, Liverpool, United Kingdom; d Malaria and Vector Resistance Laboratory, Eijkman Institute for Molecular Biology, Jakarta, Indonesia; e Mimika District Health Authority, Timika, Papua, Indonesia; f Timika Malaria Research Programme, Papuan Health and Community Development Foundation, Timika, Papua, Indonesia; g Centre for Child Health and Department of Child Health, Faculty of Medicine, Public Health and Nursing, Universitas Gadjah Mada, Yogyakarta, Indonesia; h Centers for Diseases Control and Prevention, Kisumu, Kenya; i Kenya Medical Research Institute, Centre for Global Health Research, Kisumu, Kenya; j Centers for Diseases Control and Prevention, Nairobi, Kenya; k Global and Tropical Health Division, Menzies School of Health Research, Charles Darwin University, Darwin, Northern Territory, Australia; l Centre for Tropical Medicine and Global Health, Nuffield Department of Medicine, University of Oxford, Oxford, United Kingdom

**Keywords:** dihydroartemisinin-piperaquine, population pharmacokinetic model, intermittent preventive treatment in pregnancy, nonlinear mixed-effects modeling

## Abstract

Dihydroartemisinin-piperaquine (DP) is a long-acting artemisinin combination treatment that provides effective chemoprevention and has been proposed as an alternative antimalarial drug for intermittent preventive therapy in pregnancy (IPTp). Several pharmacokinetic studies have shown that dose adjustment may not be needed for the treatment of malaria in pregnancy with DP. However, there are limited data on the optimal dosing for IPTp. This study aimed to evaluate the population pharmacokinetics of piperaquine given as IPTp in pregnant women. Pregnant women were enrolled in clinical trials conducted in Kenya and Indonesia and treated with standard 3-day courses of DP, administered in 4- to 8-week intervals from the second trimester until delivery. Pharmacokinetic blood samples were collected for piperaquine drug measurements before each treatment round, at the time of breakthrough symptomatic malaria, and at delivery. Piperaquine population pharmacokinetic properties were investigated using nonlinear mixed-effects modeling with a prior approach. In total, data from 366 Kenyan and 101 Indonesian women were analyzed. The pharmacokinetic properties of piperaquine were adequately described using a flexible transit absorption (*n* = 5) followed by a three-compartment disposition model. Gestational age did not affect the pharmacokinetic parameters of piperaquine. After three rounds of monthly IPTp, 9.45% (95% confidence interval [CI], 1.8 to 26.5%) of pregnant women had trough piperaquine concentrations below the suggested target concentration (10.3 ng/ml). Translational simulations suggest that providing the full treatment course of DP at monthly intervals provides sufficient protection to prevent malaria infection. Monthly administration of DP has the potential to offer optimal prevention of malaria during pregnancy. (This study has been registered at ClinicalTrials.gov under identifier NCT01669941 and in the ISRCTN under number ISRCTN34010937.)

## Abstract (precis)

Dihydroartemisinin-piperaquine (DP) is a long-acting artemisinin combination treatment that provides effective chemoprevention and has been proposed as an alternative antimalarial drug for intermittent preventive therapy in pregnancy (IPTp). Several pharmacokinetic studies have shown that dose adjustment may not be needed for the treatment of malaria in pregnancy with DP.

## TEXT

Compared to nonpregnant women, pregnant women are at a high risk of malaria infection and its adverse effects. Pregnant women infected with Plasmodium falciparum can develop placental malaria, with sequestration of the parasite in the placental vasculature. Placental malaria adversely affects both the mother and the infant, with adverse outcomes including maternal anemia and death, abortion, stillbirth, preterm delivery, low birth weight, infant mortality, and poor long-term child development (1). Successful malaria treatment and effective chemoprevention during pregnancy are key factors for improving maternal and child outcomes. The World Health Organization (WHO) recommends that intermittent preventive treatment in pregnancy (IPTp) with sulfadoxine-pyrimethamine (SP) should be administered at every scheduled visit during the second and third trimesters of pregnancy, spaced at least 1 month apart, to prevent the adverse consequences of malaria in pregnancy. However, there is concern that as P. falciparum resistance to SP increases, IPTp with SP will fail to provide adequate protection.

Dihydroartemisinin-piperaquine (DP) is a highly efficacious and well-tolerated antimalarial treatment. The long half-life of piperaquine provides extended malaria chemoprevention for up to 6 weeks. This antimalarial combination has therefore been suggested for malaria chemoprevention in several populations, including pregnant women. In Thai adults, a monthly dose of DP demonstrated superior protective efficacy (98% efficacy with a 95% confidence interval [CI] of 96 to 99%) compared to dosing every 2 months (86% efficacy with a 95% CI of 81 to 90%) (2, 3). Similarly, the monthly dosing of DP in school-aged children was more effective than quarterly DP or placebo (4). Seasonal malaria chemoprevention with DP in young children has efficacy against malaria similar to that of SP plus amodiaquine in areas with low parasite resistance to SP (5–7). IPTp using DP was more effective at preventing maternal and placental malaria than IPTp using SP (relative risk, 0.32 [95% CI, 0.18 to 0.56]; *P* value of <0.0001) (8–10). One of these trials also compared DP at three fixed times during pregnancy (i.e., at 20, 28, and 36 weeks of gestation) versus monthly courses of DP and showed that monthly DP exposure was associated with fewer malaria infections during pregnancy and reductions in placental parasitemia (9).

Pharmacokinetic (PK) properties in pregnancy may be altered because of several physiological changes, including increased water and fat contents, increased renal function, and altered enzymatic expression and degree of plasma protein binding. For example, exposures to artemether, artesunate, chloroquine, dihydroartemisinin, lumefantrine, sulfadoxine, pyrimethamine, atovaquone, proguanil, and cycloguanil are altered during pregnancy (11–16). In contrast, exposures to quinine and amodiaquine are unaffected by pregnancy (17, 18). Therefore, pharmacokinetic investigation in pregnancy is needed to determine if any alterations exist and to optimize the dosing regimen in pregnant women.

Pharmacokinetic properties of piperaquine as part of case management (i.e., treatment) of women with malaria have been investigated in several pregnant populations (19–25). Several studies reported unaltered piperaquine exposure (area under the concentration-time curve [AUC]) in pregnant women (19–22), while another study reported that exposure was 40% lower in pregnant women than in nonpregnant women (23). However, one of these studies (20), reporting similar exposures in pregnant and nonpregnant women, still presented altered pharmacokinetic properties in pregnant women (i.e., matched increase in elimination clearance and relative bioavailability, resulting in unchanged total exposure). While the population pharmacokinetics of piperaquine have been extensively evaluated in the treatment of acute malaria in pregnant women (19–23, 26), pharmacokinetic properties of piperaquine in monthly IPTp (i.e., chemoprevention) are largely unreported. One previously reported IPTp study reported a 72% higher elimination clearance rate in pregnant women than in postpartum women, resulting in a substantially lower total exposure to piperaquine (25). Increased elimination clearance of piperaquine in pregnant women, reported in both acute treatment and IPTp, would have a substantial impact on the total exposure and trough concentrations achieved with repeated monthly IPTp. A recent study evaluating piperaquine in IPTp suggested optimal piperaquine target concentrations of 10.9 ng/ml and 13.9 ng/ml, associated with 95% and 99% protective efficacies, respectively, against P. falciparum infections during pregnancy (24). The study presented here aimed to describe the population pharmacokinetic properties of piperaquine in pregnant women receiving IPTp with DP.

## RESULTS

The main results of these two clinical trials have been reported previously (8, 27). Part of the IPTp arm from these two clinical trials provided pharmacokinetic samples, including 366 Kenyan pregnant women and 101 Indonesian pregnant women. Full demographic characteristics are presented in Table 1.

**TABLE 1:** Demographic parameters and treatment outcomes of pregnant women

Parameter	Value for group	
	Kenya site	Indonesia site
Total no. of pregnant women	366	101
Total no. of pharmacokinetic samples^*a*^	1,213	342
Median total monthly dose of piperaquine base (mg/kg) (range)	25.7 (13.8–34.7)	29.3 (21.1–41.3)
Continuous and categorical covariates at admission		
Median age (yrs) (range)	23.0 (14.0–42.0)	27.0 (16.0–41.0)
Median body wt (kg) (range)	60.9 (44.5–112)	52.6 (37.4–79.8)
Median corrected gestational age at admission (wks) (range)	22.9 (7.57–35.7)	25.0 (14.6–33.0)
No. (%) of pregnant women with placental malaria outcome (no. of women with outcome/total no. tested)		
Past infection by histology	27.7 (97/350)	0.00 (0/89)
Chronic infection by histology	1.14 (4/350)	2.25 (2/89)
Acute infection by histology	0.857 (3/350)	0.00 (0/89)
Any placental malaria (RDT, smear, PCR, or histology)^*b*^	31.7 (112/353)	3.26 (3/92)

a Pregnant women from Kenya provided venous plasma samples, and pregnant women from Indonesia provided capillary dried blood spot samples.

b RDT, rapid diagnostic test.

### Pharmacokinetic properties of piperaquine.

**FIG 1:**
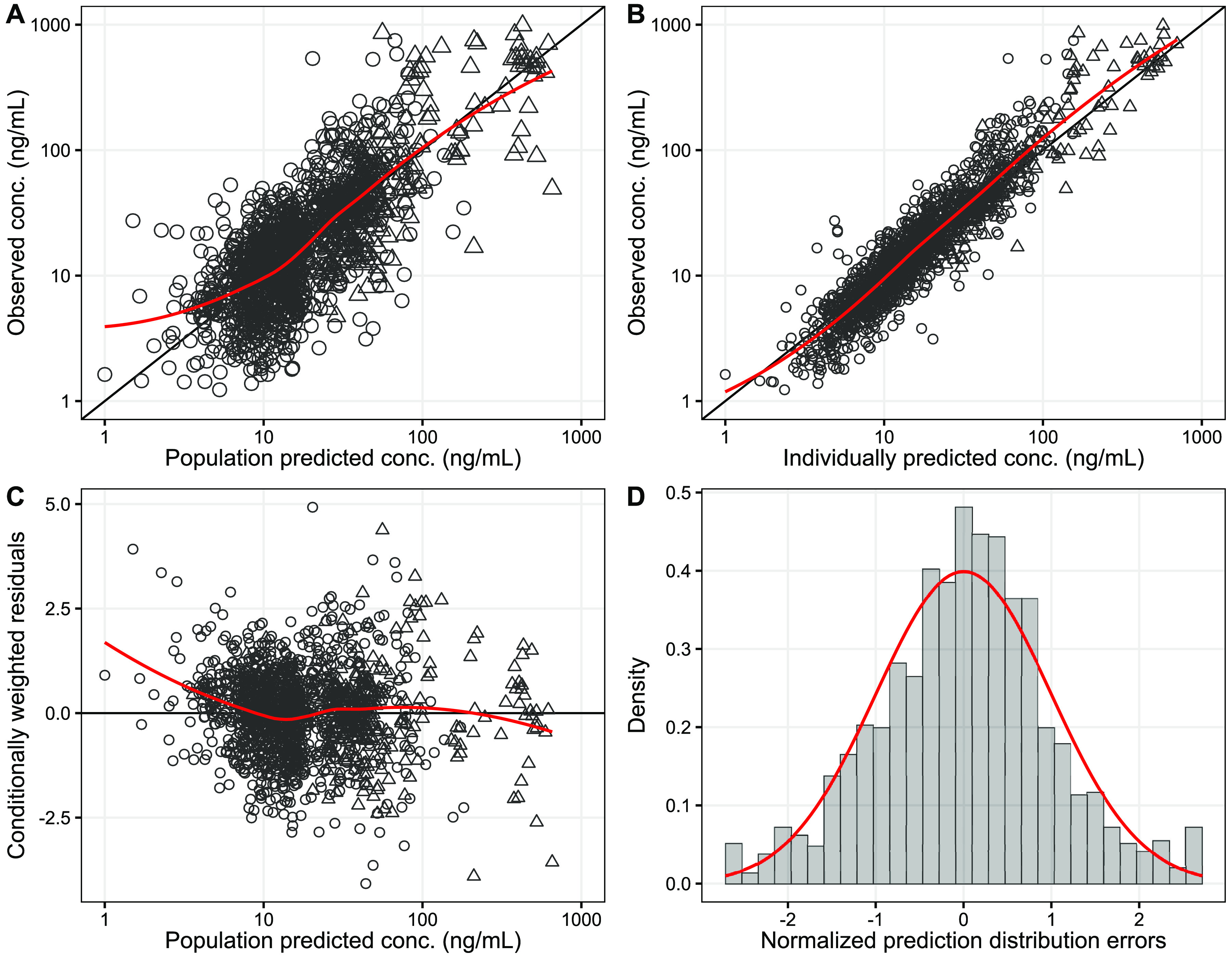
Goodness of fit of piperaquine, stratified by study sites. (A) Population predictions versus observations; (B) individual predictions versus observations; (C) population predictions versus conditionally weighted residual errors; (D) distribution of the normalized prediction distribution errors. Open circles are venous plasma concentrations, and open triangles are capillary dried blood spot concentrations. Solid lines represent locally weighted least-squares regressions, based on both capillary and venous data.

**FIG 2:**
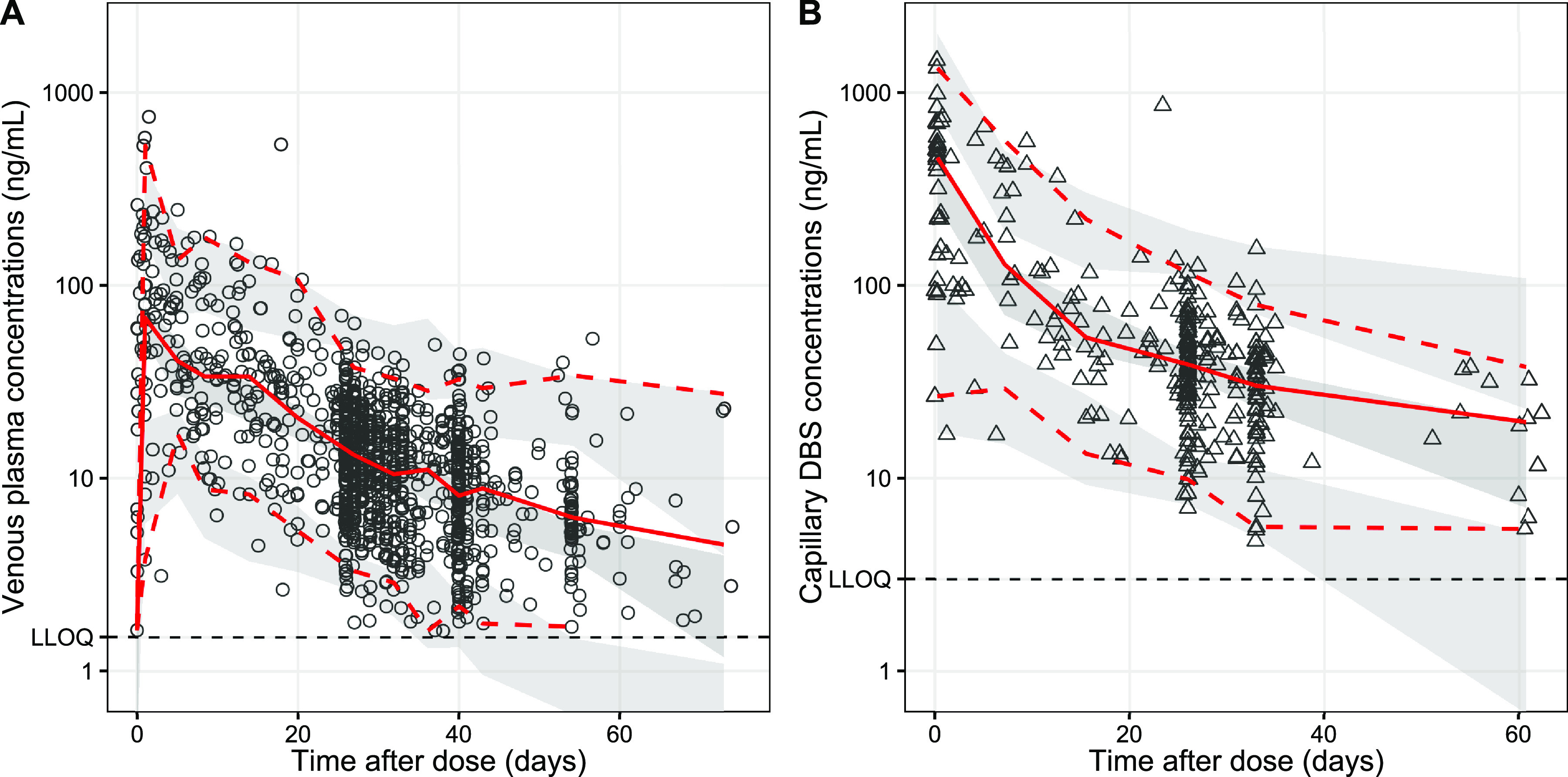
Visual predictive plots of piperaquine in pregnant women in Kenya (A) and Indonesia (B). Open markers represent observed concentrations. Solid and dashed lines represent the median and the 5th and 95th percentiles of the observations. Shaded areas represent the predicted 95% confidence intervals of each percentile.

**FIG 3:**
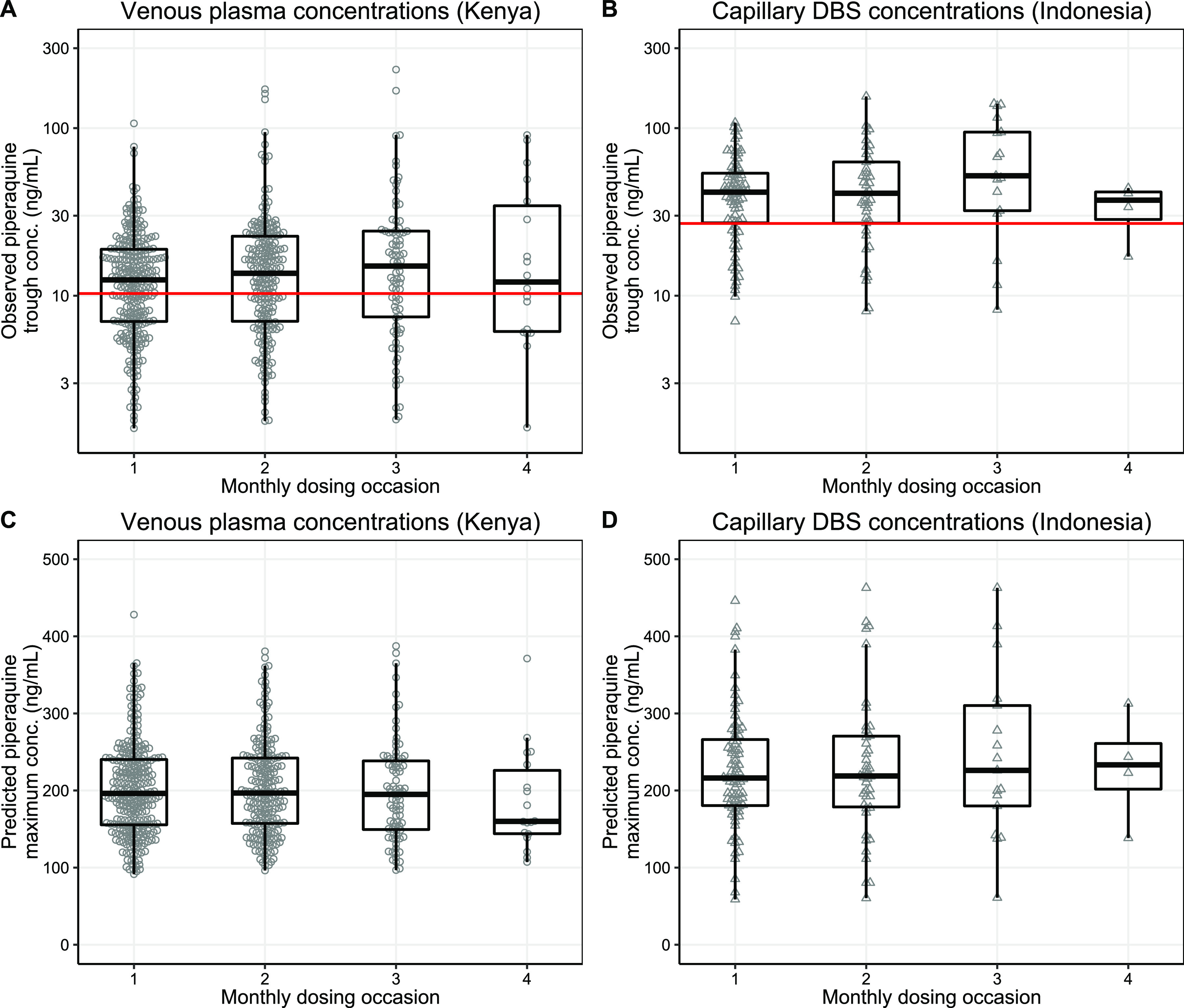
Observed piperaquine trough concentrations (*C*_min_) and predicted piperaquine maximum concentrations (*C*_max_). (A) Plasma trough concentrations in Kenyan pregnant women; (B) capillary DBS trough concentrations in Indonesian pregnant women; (C) plasma piperaquine maximum concentrations in Kenyan pregnant women; (D) capillary DBS piperaquine maximum concentrations in Indonesian women. The box-and-whisker plots represent the medians with interquartile ranges and the 95% prediction intervals. The horizontal red lines represent a target piperaquine plasma concentration in pregnant women of 10.3 ng/ml (equivalent to 26.9 mg/ml capillary DBS concentrations) (24).

The population pharmacokinetic properties of piperaquine were described successfully using a prior approach with a model developed previously (20). The final model showed satisfactory goodness of fit (Fig. 1) and predictive performance, as illustrated by the visual predictive check (Fig. 2). High eta shrinkages were seen in the final model (i.e., more than 30%) because of the sparseness of the observations, but the epsilon shrinkage was low (20.0% for plasma samples and 22.1% for dried blood spot [DBS] samples). Allometrically scaled body weight was implemented into the pharmacokinetic model, according to the prior model (20). Pregnancy-related parameters (corrected gestational age as a time-varying covariate) or other admission covariates did not exhibit a significant impact on the pharmacokinetic parameters of piperaquine in this study. Final pharmacokinetic parameter estimates are summarized in Table 2, and secondary pharmacokinetic parameters are summarized in Table 3. Piperaquine peak concentrations (*C*_max_) for the Kenyan and Indonesian pregnant women were predicted to be 212 ng/ml (95% CI, 144 to 319 ng/ml) and 232 ng/ml (95% CI, 108 to 396 ng/ml), respectively. Observed trough piperaquine concentrations accumulated substantially with repeated monthly IPTp, but predicted peak concentrations remained similar during the entire duration of IPTp (Fig. 3).

**TABLE 2:** Final population pharmacokinetic parameters^*a*^

Parameter	Prior estimate^*b*^	Population estimate^*c*^	95% confidence interval^*d*^	%RSE^*d*^
Pharmacokinetic parameter estimates				
MTT (h)	2.04	2.10	1.90–2.30	4.87
CL/*F* (liters/h)	59.4	49.0	47.0–51.2	2.17
*V_C_*/*F* (liters)	2,140	2,190	1,800–2,560	8.91
*Q*_1_/*F* (liters/h)	276	244	200–294	9.97
*V_P_*_1_/*F* (liters)	3,560	3,270	2,460–4,100	13.3
*Q*_2_/*F* (liters/h)	105	98.2	79.3–212	11.0
*V_P_*_2_/*F* (liters)	20,700	18,800	17,500–20,200	3.65
CF_CAP-VEN_	NA	2.62	2.35–2.87	5.18
*F*	NA	1 (fixed)	NA	NA
σ_CP_	NA	0.291	0.257–0.329	3.16
σ_DBS_	NA	0.229	0.173–0.290	6.45

Interindividual variability (%CV)				
CL/*F* (liters/h)	19.6	21.0	0.0385–0.0502	3.42
*V_C_*/*F* (liters)	38.5	38.9	0.117–0.169	4.75
*Q*_2_/*F* (liters/h)	34.6	36.0	0.0895–0.175	9.03

Interoccasion variability (%CV)				
MTT (h)	36.0	36.2	0.109–0.140	3.24
*F*	41.1	41.5	0.138–0.193	6.45

a Abbreviations: CL, elimination clearance; CF_CAP-VEN_, proportional conversion factor between capillary and venous drug measurements; *F*, relative bioavailability; MTT, mean absorption transit time; *Q*, intercompartment clearance; σ_CP_, variance of proportional residual errors of plasma samples; σ_DBS_, variance of proportional residual errors of dried blood spot samples; *V_C_*, central volume of distribution; *V_P_*, peripheral volume of distribution. NA, not applicable.

b The final model and parameter estimates of the pharmacokinetic study of piperaquine in pregnant women (20) were used as a frequentist prior.

c Computed population mean parameter estimates from NONMEM were calculated for a typical pregnant woman at a body weight of 48.5 kg. The percent coefficient of variation (%CV) for interindividual variability was calculated as $\sqrt{\text{exp}\left({\text{ω}}^{2}\right)-1}\times 100$.

d Computed from the sampling important resampling (SIR) procedure (45, 46) of the final pharmacokinetic model with 6 iterations of 1,000, 1,000, 1,000, 2,000, 2,000, and 2,000 samples and 200, 200, 400, 500, 500, and 500 resamplings.

**TABLE 3:** Secondary pharmacokinetic parameters after the first round of IPTp using DP^*a*^

Parameter	Median value (range) for group	
	Kenya site (plasma samples)	Indonesia site (DBS samples)
*C*_max_ (ng/ml)	212 (144–319)	232 (108–396)
*T*_max_ (h)	2.10 (2.06–2.15)	2.07 (2.01–2.14)
Terminal half-life (days)	20.2 (16.2–24.7)	19.4 (15.5–23.4)
Day 7 plasma concn (ng/ml)	30.1 (17.2–50.6)	32.4 (14.0–54.5)
Day 28 plasma concn (ng/ml)	12.7 (5.80–151)	37.5 (13.4–296)
AUC_28 day_ (ng · h/ml)	20,400 (12,400–32,600)	22,200 (9,550–36,800)

a Abbreviations: AUC_28 day_, area under the concentration-time curve up to 28 days; *C*_max_, maximum concentration; DBS, dried blood spot; *T*_max_, time to maximum concentration.

### Implication for placental malaria.

**FIG 4:**
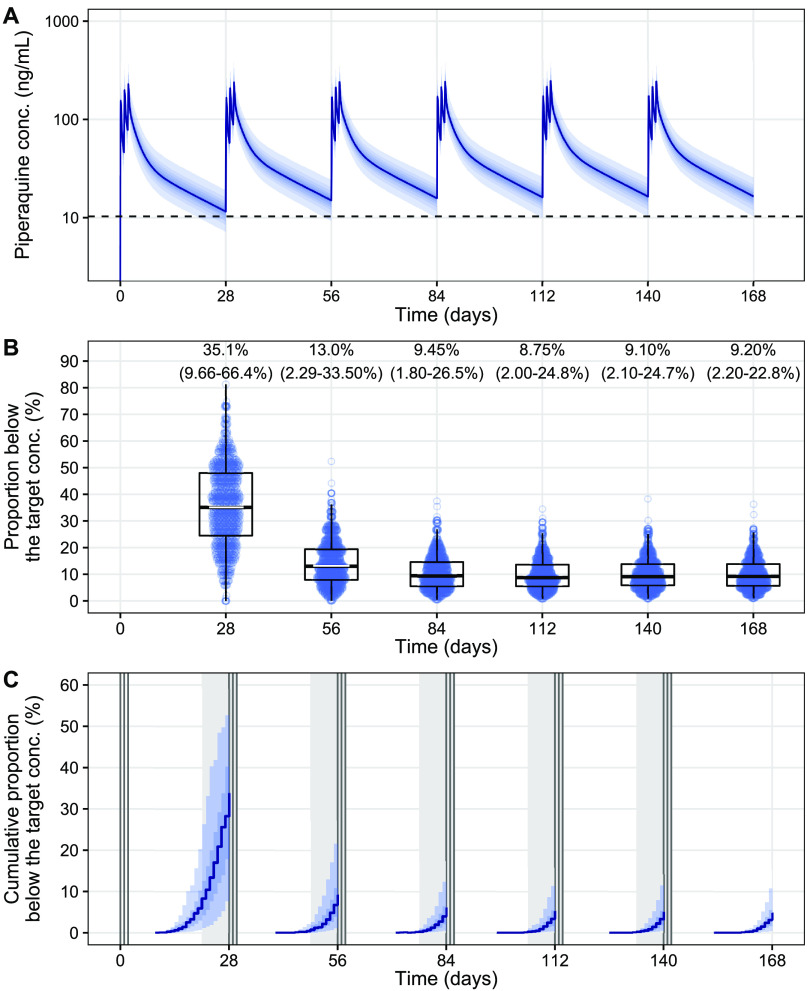
Simulation of monthly piperaquine dosing in pregnant women receiving IPTp. (A) Venous piperaquine plasma concentrations versus time (*n* = 1,000). The blue solid line represents the predicted median piperaquine concentration-time profile, and the shaded area represents the 95% prediction interval. The horizontal dashed black line represents the proposed target plasma concentration of 10.3 ng/ml (24). (B) Proportion of patients with piperaquine trough concentrations below the target concentration (*n* = 1,000 individuals; 1,000 replicates). The box-and-whisker plots represent the medians with interquartile ranges and the 95% prediction intervals of the simulated concentrations. (C) Cumulative proportion of patients with plasma concentrations below the target concentrations (10.3 ng/ml) (blue solid lines) and 95% prediction intervals (blue shaded areas). The triple vertical lines represent monthly dosing. The gray-shaded areas represent a 1-week time interval, preceding a dose in which submicroscopic infection can be eliminated by the next IPTp treatment round.

Placental malaria during delivery was assessed by either a rapid diagnostic test (RDT), blood smear, placental blood PCR, or placental tissue histology and was detected in 3.3% (3/92) of Indonesian women and 31.7% (112/353) of Kenyan women (Table 1). However, 27.7% (97/350) of placental malaria infections in Kenyan women were past infections (i.e., malaria pigment present but no malaria parasites visible). Only 0.9% (3/350) and 1.14% (4/350) of Kenyan woman presented with acute and chronic infections, respectively. Therefore, the small number of observed active placental malaria cases was not sufficient to undertake statistical analysis or pharmacodynamic (PD) modeling. Translational simulations of the final pharmacokinetic model were conducted to illustrate the possibility of patients having subtherapeutic concentrations (Fig. 4). Based on the reported target trough piperaquine concentrations of 10.3 ng/ml to prevent P. falciparum infection during pregnancy (24), simulations predicted that approximately 35.1% (95% CI, 9.66% to 66.4%), 13.0% (95% CI, 2.29% to 33.5%), and 9.45% (95% CI, 1.80% to 26.5%) of individuals had trough concentrations below the target concentration after the first, second, and third rounds of IPTp, respectively. The piperaquine plasma concentrations became subtherapeutic within 1 week prior to the next IPTp dose; hence, new infections acquired within this time window (i.e., 1 week before the next IPTp dose) are unlikely to develop into a clinical symptom since there is insufficient time for the malaria parasites to replicate to the level of symptomatic parasitemia (biomass of ∼10^8^ parasites) before the subsequent IPTp dose if taken monthly (Fig. 4).

## DISCUSSION

Our analysis highlights that a standard 3-day treatment course of DP, provided monthly as IPTp, appears to provide sufficient protection from malaria infection in pregnant women. This finding was apparent in both Kenya and Indonesia, with only 7 of 350 pregnancies presenting with placental malaria infection at delivery. An estimated 90.6% (95% CI, 73.5 to 98.2%) of women are likely to maintain piperaquine trough concentrations above 10.3 ng/ml, and 66.8% (95% CI, 64.0 to 70.5%) are likely to maintain concentrations above 13.9 ng/ml, concentration thresholds previously found to be associated with 95% and 99% malaria protection after three rounds of DP dosing, respectively (24). However, these therapeutic target concentrations might need to be substantially higher in areas of emerging drug resistance to DP due to reduced drug susceptibility (28, 29).

Even though our analysis utilized samples collected from two studies and almost 500 recruited pregnant women (>1,500 samples), most samples were collected close to trough concentrations, resulting in insufficient data to develop a robust absorption and distribution model of piperaquine. Piperaquine pharmacokinetics are normally described using a multiphasic disposition model and transit compartment absorption (20, 22, 24, 25, 30–32). To overcome this limitation, we applied a frequentist prior approach from a pharmacokinetic study of piperaquine in Thai pregnant women with a rich sampling design (20). The final estimates of absorption parameters (median absorption transit time [MTT] and interoccasion variabilities on MTT and *F*) relied heavily on the prior model, resulting in estimated 95% confidence intervals including the prior estimates. On the other hand, the clinical trial data were informative in determining the elimination clearance and predicting the trough concentrations in this study, resulting in a significantly different clearance in this study compared to that of the prior study (i.e., the 95% confidence interval of the parameter estimate did not include the prior value).

This study did not recruit nonpregnant patients, and therefore, it was not possible to determine if, overall, pregnancy had an effect on the pharmacokinetic properties of piperaquine. The potential effects of pregnancy on the pharmacokinetic properties of piperaquine are still unclear. Several previous studies have shown that pregnancy has no effect on the pharmacokinetic properties of piperaquine (19, 22). However, one study showed that pregnant patients showed 45% higher clearance and 47% lower absorption than nonpregnant women, resulting in similar drug exposures in the two groups (20). A noncompartmental pharmacokinetic study in Sudanese pregnant and nonpregnant patients reported significantly higher piperaquine exposure in pregnant women after the first dose, but the total piperaquine exposure was not different between the two groups (21). A recent IPTp study showed that pregnant women had substantially lower exposure to piperaquine than postpartum women (i.e., 72% higher clearance) (25). HIV-infected pregnant women on efavirenz-based antiretroviral treatment and pregnant women with a low body mass index in the study also had altered pharmacokinetic properties of piperaquine. Furthermore, we saw no statistically significant effect of gestational age on pharmacokinetic parameters when it was evaluated as a time-varying covariate in these pregnant patients. This finding was supported by a previous study reporting no difference in the elimination clearance rates between the second and third trimesters (22). This suggests that the same dose regimen could be maintained for the duration of IPTp. Pharmacokinetic parameters were also scaled allometrically by body weight based on the strong biological prior of such a covariate and the reported literature supporting this relationship (20, 22, 30, 32, 33). Other baseline covariates had no significant impact on any piperaquine pharmacokinetic parameters and were not retained in the final model. The final pharmacokinetic parameter estimates are in agreement with those of previous pharmacokinetic studies (30, 33).

Venous plasma concentrations from the Kenyan pregnant women and the capillary dried blood spot concentrations from the Indonesian pregnant women were modeled simultaneously using a population conversion factor (CF_CAP-VEN_). Since the different study sites provided different samples (venous plasma versus capillary dried blood spots), the population estimates of the conversion factor between capillary dried blood spot concentrations and venous plasma concentrations might be interpreted as a combination of sampling differences, ethnicity differences, and other unknown site-specific differences. However, previously reported results have not shown any evidence of clinically important ethnic differences associated with the pharmacokinetic properties of the nine antimalarial drugs used in the standard artemisinin-based combination therapy (ACT) regimens recommended by the WHO (34). Furthermore, the estimated conversion factor was in agreement with previous estimates from studies with both plasma and capillary measurements in each patient (5, 33).

Piperaquine samples were not collected at the time of peak concentrations in this study. Thus, the estimated peak piperaquine concentrations were influenced mainly by the prior model (i.e., the prior estimates of the absorption parameters). Peak piperaquine concentrations were estimated to be approximately 17-fold higher than trough concentrations. Therefore, the remaining piperaquine concentrations at the end of the monthly round, associated with the accumulation of piperaquine trough concentrations, had a very minor impact on the peak piperaquine concentrations due to the relatively small contribution to total peak concentrations (Fig. 3C and D). Piperaquine is associated with concentration-dependent corrected QT interval (QTc) prolongation, resulting in the greatest risk of QTc prolongation during peak concentrations, which occur approximately 4 to 6 h after the third dose of DP during each course of treatment (30, 35, 36). However, electrocardiogram (ECG) was performed on a small subset of pregnant patients (*n* = 33) in Indonesia and did not show any increase in absolute QTc or QTc prolongation with repeated cycles of monthly DP dosing (27). This supports further the modeling results showing no substantial accumulation in estimated peak piperaquine concentrations with repeated monthly dosing of DP. Even so, a pharmacokinetic-electrocardiographic study of IPTp-DP in pregnant women is needed to evaluate the cardiac safety of piperaquine.

Monthly dosing of DP provides better protection against malaria than less frequent dosing (2, 3, 24, 37). None of the Indonesian patients presented with active placental malaria at delivery, and only 7 out of 350 Kenyan pregnant women who received DP at 4- to 6-week intervals presented active placental malaria. Due to the small number of women with placental malaria, we were unable to determine an exposure-response relationship directly. Thus, we relied on translational simulations to determine the success of the pharmacokinetic outcome. Using a suggested target venous plasma piperaquine concentration of 10.3 ng/ml, associated with 95% protection from P. falciparum infection (24), approximately 90.6% (95% CI, 73.5 to 98.2%), 91.3% (95% CI, 75.2 to 98.0%), and 90.8% (95% CI, 77.2 to 97.8%) of pregnant women reached the target trough concentration after three, four, and five rounds of monthly dosing, respectively. However, in the women who did not achieve the target trough concentration, the piperaquine concentrations dropped below this target level only just before the next monthly IPTp dose, suggesting that P. falciparum infections acquired during this period would have been treated with the subsequent round of DP. Thus, monthly IPTp dosing of DP was concluded to be appropriate for pregnant women living in areas where malaria is endemic. Dosing adjustment in pregnant women in the first round of IPTp would be desirable in order to maintain trough concentrations above the target level. However, several arguments indicate that changing DP dosing in the first round of IPTp might be impractical. An increased DP dose during the first round of IPTp would generate a proportional increase in peak concentrations, which could result in safety concerns (i.e., QTc prolongation). An altered administration schedule during the first round of IPTp might lead to poor drug adherence. The most important aspect of preventive treatment is adherence, and pregnant women are scheduled to visit the antenatal care (ANC) clinic on a monthly basis. The monthly dosing regimen is the most practical way to administer these preventive treatments and is likely to result in high efficacy when taken as instructed. Thus, adherence to the full 3-day course of DP is the main concern, and missing any of the home-administered doses (2nd and/or 3rd dose) will result in subtherapeutic piperaquine concentrations for a substantial duration of time before the next round of IPT dosing.

This study has several limitations. IPTp is recommended for pregnant women during the 2nd and 3rd trimesters (38). Pregnant women in this pregnancy period have major physiological differences compared to nonpregnant women and women in the first trimester of pregnancy. All participants in this study were in their 2nd and 3rd trimesters of pregnancy. Thus, this study had limited power to detect possible effects of gestational age on the pharmacokinetic properties of piperaquine and no possibility to detect possible differences between pregnant and nonpregnant women. Different types of sampling methodologies were applied in the two study sites (i.e., venous plasma versus dried capillary blood on filter paper). Therefore, the conversion between venous and capillary concentrations could not be estimated within a patient, and we cannot exclude that the population-estimated conversion factor includes several confounding study-specific effects (e.g., matrix effects, ethnic differences, demographic study differences, and/or unknown study differences). Only piperaquine trough concentrations were sampled in this study. Therefore, the final pharmacokinetic model structure and its parameter estimates relied heavily on the prior model and the observations during the elimination phase. Especially absorption and early distribution parameter estimates in the final model, where the observations were limited, were very much influenced by the prior model. However, the piperaquine trough concentration is the main determinant of successful preventive treatment and, therefore, the main clinical interest in this study.

### Conclusions.

The population pharmacokinetic properties of piperaquine were successfully evaluated in pregnant women receiving IPTp. Five transit compartments followed by a three-compartment disposition model described the pharmacokinetic properties of piperaquine adequately. Gestational age and other baseline covariates had no significant effect on the pharmacokinetic properties of piperaquine. Modeling and simulation suggested that more than 90.3% of pregnant women who receive three monthly courses of IPTp achieved piperaquine exposures associated with protection against acquired malaria infections. Predicted peak concentrations did not accumulate with repeated dosing courses, suggesting that IPTp with DP is not likely to increase the risk for QT interval prolongation associated with piperaquine exposure, but further cardiac safety data are still needed. The PK/PD analysis presented here suggested that monthly IPTp with DP is likely to be highly protective against placental malaria.

## MATERIALS AND METHODS

### Study design.

This population pharmacokinetic study included pregnant women from two distinct randomized three-arm clinical trials. Both trials included intermittent screening and treatment in pregnancy (ISTp) with DP (“ISTp-DP”) and IPTp with DP (“IPTp-DP”). In Kenya, the third arm consisted of IPTp with SP, and in Indonesia, this was single screening and treatment with DP (SSTp-DP) (8, 27). Both studies included pharmacokinetic samplings in the IPTp-DP and ISTp-DP arms and were included in this population pharmacokinetic analysis.

All pregnant women received a 3-day fixed oral combination of dihydroartemisinin and piperaquine (Eurartesim; Sigma-Tau, Pomezia, Italy) (40 mg dihydroartemisinin and 320 mg piperaquine tetraphosphate per tablet), dosed by weight at enrollment according to the manufacturer’s recommendations, approximately once a month in the Indonesian study and at 4- to 6-week intervals in the Kenya study (8, 27). The first day of administration of each month was supervised, and the date and time of the administrations were recorded and used for further pharmacokinetic analysis. The consecutive doses were taken unsupervised at home. However, health care workers visited all participants at home to confirm adherence to the drug regimen. All confirmed malaria cases in Kenya during follow-up were treated with artemether-lumefantrine (Coartem). The confirmed recurrent malaria cases in Indonesia were treated with quinine-clindamycin (10 mg/kg of body weight twice daily for 7 days). Their data, after the time of recurrent malaria, were excluded from the pharmacokinetic analysis.

### Blood sampling.

In Kenya, a baseline venous blood sample was collected from all women prior to antimalarial administration. Trough venous samples (1.5 ml) were collected before each monthly drug administration. An additional venous sample was collected at the time of delivery. All blood samples were centrifuged (2,000 × *g* for 10 min), and plasma samples were stored at −80°C until shipment on dry ice. In Indonesia, capillary samples were collected by finger prick according to the same schedule. A drop of blood was collected on filter paper (31ETCHR; Whatman), and the filter paper was dried horizontally (1 h at 50% humidity) and packed into separate plastic bags with silica gel. The plasma samples from Kenya and dried blood spot samples from Indonesia were transported for drug analysis to the Department of Clinical Pharmacology, Mahidol-Oxford Tropical Medicine Research Unit (MORU), Bangkok, Thailand.

### Drug quantification.

Piperaquine plasma concentrations were measured using solid-phase extraction followed by liquid chromatography coupled with tandem mass spectrometry according to a previously reported method (39). Quality control (QC) samples at 4.50, 20.0, and 400 ng/ml were analyzed in triplicate within each batch of clinical samples to ensure the accuracy and precision of the assay. The percent relative standard deviations (%RSDs) at low, middle, and high concentration levels were 4.70%, 4.38%, and 4.92%, respectively. The limit of detection (LOD) and the lower limit of quantification (LLOQ) were set to 0.375 and 1.50 ng/ml, respectively. Piperaquine dried blood spot concentrations were measured using solid-phase extraction according to a previously reported method, with modification (40), followed by liquid chromatography coupled with tandem mass spectrometry (39). QC samples at 9.00, 40.0, and 800 ng/ml were analyzed in triplicate within each batch of clinical samples to ensure the accuracy and precision of the assay. The %RSDs at low, middle, and high concentration levels were 4.36%, 3.33%, and 3.94%, respectively. The LOD and the LLOQ were set to 1 and 3 ng/ml, respectively. The laboratory where testing occurred is a participant in the quality assurance/quality control (QA/QC) proficiency testing program supported by the WorldWide Antimalarial Resistance Network (WWARN) (41).

### Population pharmacokinetic analysis.

Observed piperaquine concentrations were logarithmically transformed and analyzed using nonlinear mixed-effects modeling using NONMEM version 7.4 (Icon Development Solutions, Ellicott City, MD). Pirana version 2.9.0 (42), Perl-speaks-NONMEM version 4.7.0 (PsN) (43), and R version 3.4.4 were used for automation, model evaluation, and diagnostics during the model-building process. The first-order conditional estimation method with interactions (FOCE-I) was used throughout the population pharmacokinetic analysis. The proportion of measured drug concentrations below the LLOQ was low (2.47% in total) and therefore omitted from further pharmacokinetic analysis. The $PRIOR functionality in NONMEM was used to stabilize the model performance. A previously reported population pharmacokinetic model, describing DP in the treatment of uncomplicated falciparum malaria in pregnant women in Thailand, was used as the prior model (20). The structural model of piperaquine included five transit absorption compartments followed by a three-compartment disposition model. Final population pharmacokinetic parameter estimates, between-patient variability estimates, and between-occasion variability estimates with their uncertainties were implemented as priors.

Pharmacokinetic parameters were assumed to be log-normally distributed and therefore implemented as exponential between-patient and between-occasion variabilities as Θ*_ij_* = Θ × exp(η*_i_*_,Θ_ + κ*_ij_*_,Θ_), where Θ*_ij_* is the pharmacokinetic parameter estimate for the *i*th patient at the *j*th occasion, Θ*_ij_* is the typical pharmacokinetic parameter estimate of the population, η*_i_*_,Θ_ is the between-patient variability of parameter Θ in the *i*th patient, and κ*_ij_*_,Θ_ is the between-occasion variability of parameter Θ in the *i*th patient at the *j*th occasion. Both between-patient variability and between-occasion variability were assumed to be normally distributed with a zero mean and ω^2^ variance. Estimated between-patient variability below 10% or variability estimated with poor precision (i.e., %RSE > 50%) was fixed to zero. Residual unexplained variability was modeled as an additive error on the log-transformed observed concentrations, which is essentially equivalent to an exponential error on an arithmetic scale.

Individual body weight (BW*_i_*) was measured at enrollment only and introduced into the pharmacokinetic model as a fixed allometric function on all volume (exponent of *n* = 1.00) and clearance (exponent of *n* = 0.75) parameters, scaled to the median body weight (48.5 kg) of the prior study population, as follows: Θ*_i_* = Θ × exp(η*_i_*_,Θ_ + κ*_ij_*_,Θ_) × (BW*_i_*/48.5)*^n^*.

The population conversion factor (CF) between venous plasma concentrations and capillary blood concentrations was estimated without between-patient variability. All other covariates (i.e., corrected gestational age, maternal age, and sex) were investigated by a stepwise addition (*P* < 0.05) and elimination (*P* < 0.001) approach. The corrected gestational age was implemented as a time-varying covariate. The effect of gestational age was also modeled separately using a full covariate approach in which the gestational age was implemented as a continuous covariate on all pharmacokinetic parameters in the final pharmacokinetic model. Secondary pharmacokinetic parameter estimates were derived from the *post hoc* pharmacokinetic parameter estimates of the final pharmacokinetic model.

### Model diagnostics and evaluations.

Model fitness was evaluated primarily by the objective function value (OFV) (calculated by NONMEM as proportional to −2× log likelihood of the data). Model discrimination between two hierarchical models was determined by a likelihood ratio test, based on the chi-square distribution of the OFV (i.e., *P* value of <0.05 is equivalent to ΔOFV of <−3.84, at 1 degree of freedom difference). Potential model misspecification and systematic errors were evaluated by basic goodness-of-fit diagnostics. Eta and epsilon shrinkages were used to assess the ability to detect model misspecifications in goodness-of-fit diagnostics (44). Model robustness and parameter confidence intervals were evaluated by a sampling important resampling (SIR) procedure (45, 46). Predictive performances of the final models were illustrated by prediction-corrected visual and numerical predictive checks (*n* = 2,000) (47). The 5th, 50th, and 95th percentiles of the observed concentrations were overlaid with the 95% confidence intervals of each simulated percentile to detect model bias.

### Translational simulations.

The final population pharmacokinetic model was used to simulate a population pharmacokinetic profile of monthly piperaquine IPTp in 1,000 pregnant women in 1,000 hypothetical clinical trials. The previously suggested target piperaquine concentration of 10.3 ng/ml, which provided 95% protection from P. falciparum infection during pregnancy in a previous IPTp study in Uganda (24), was considered the pharmacokinetic outcome target. The proportions of pregnant women with trough piperaquine plasma concentrations below/above the target concentrations were simulated at each of the monthly doses.

### Ethical approval.

All procedures performed in studies involving human participants were in accordance with the ethical standards of the institutional and/or national research committee and with the 1964 Helsinki declaration and its later amendments or comparable ethical standards. Ethical approval for the Kenyan study was obtained from the Kenya Medical Research Institute and the U.S. Centers for Disease Control and Prevention. This study was registered with ClinicalTrial.gov under identifier NCT01669941. Ethical approval for the Indonesian study was obtained from Liverpool School of Tropical Medicine (12.28), Eijkman Institute for Molecular Biology (project N:57), and Litbangkes, Ministry of Health, Jakarta (LB02.01/5.2/KE059/2013). This clinical trial was registered at the ISRCTN registry under number ISRCTN34010937. Written informed consent was obtained from all participants.
